# Digitale Behandlung der Fibromyalgie

**DOI:** 10.1007/s00393-025-01738-5

**Published:** 2025-10-24

**Authors:** Thomas Hügle

**Affiliations:** Abteilung Rheumatologie, Universitätsspital Lausanne (CHUV), Universität Lausanne, Avenue Pierre-Decker 4, 1011 Lausanne, Schweiz

**Keywords:** Digitale Gesundheitsanwendung, Selbstmanagement, Kognitive Verhaltenstherapie, Mobile Health, Apps, Digital health application, Self-management, Cognitive behavioral therapy, Mobile health, Apps

## Abstract

**Hintergrund:**

Fibromyalgie ist ein chronisches Schmerzsyndrom mit erheblicher körperlicher und psychischer Belastung. Die Wirksamkeit medikamentöser Therapien ist begrenzt, während nichtmedikamentöse Selbstmanagementstrategien wie Bewegung, kognitive Verhaltenstherapie (KVT) und achtsamkeitsbasierte Verfahren effizient sind und empfohlen werden, aber nur begrenzt zum Einsatz kommen. Diese lassen sich zunehmend auch online durchführen, was den Zugang erleichtert.

**Ziel der Arbeit:**

Ziel dieses Beitrags ist es, auf Basis des aktuellen Stands zu prüfen, wie Selbstmanagementansätze bei der Fibromyalgie digital über Apps umgesetzt werden und wie digitale Therapien z. B. mittels Wearables, KI(künstliche Intelligenz)-Modellen und Chatbots weiterentwickelt werden können.

**Methoden:**

Bestehende digitale Anwendungen für Fibromyalgie und neue Konzepte wurden bewertet. Exemplarisch wurde eine eigene entwickelte App POCOS (Patient Organiser and Companion System) mit und ohne KI-Chatbot in Fokusgruppen mit Patient:innen diskutiert.

**Ergebnisse:**

Zunehmend erhältliche digitale Anwendungen für Fibromyalgie decken bislang primär psychoedukative Inhalte ab, während Bewegungsprogramme und multimodale Konzepte seltener integriert sind, aber evidenzbasiert sind und von Patient:innen gewünscht werden. Klinische Studien konnten einen positiven Effekt einzelner Online-Programme nachweisen. Fokusgruppen plädieren für eine personalisierte, niedrigschwellige und benutzerfreundliche Gestaltung der Apps. Der Einsatz von KI-gestützten Chatbots in diesem Kontext ist möglich und sinnvoll, bedarf jedoch strukturierter Inhalte und regulatorischer Rahmenbedingungen. Auf diagnostischer Ebene können durch maschinelles Lernen „Fibromyalgietypen“ identifiziert werden, die hilfreich für eine personalisierte Behandlung sind.

**Diskussion:**

Die digitale Behandlung der Fibromyalgie ist möglich und wirksam, erfordert aber eine konsequente evidenzbasierte Umsetzung, Partizipation von Betroffenen, technische Interoperabilität und eine sensible Gestaltung für die heterogene Zielgruppe.

## Abstract (en)

**Background:**

Fibromyalgia is a chronic pain syndrome associated with a substantial physical and psychological burden. The effectiveness of pharmacological treatment is limited, whereas nonpharmacological self-management strategies, such as exercise, cognitive behavioral therapy (CBT) and mindfulness-based approaches, are recommended. These can increasingly be delivered as online programs and significantly improve access to these forms of treatment.

**Objective:**

Based on the current evidence, the aim of this article is to review how self-management approaches for fibromyalgia are digitally implemented via apps and how these could be further developed using, for example, wearables, artificial intelligence (AI) models and chatbots.

**Methods:**

Existing digital applications for fibromyalgia and novel concepts were evaluated. As an example, the authors’ own app, the patient organiser and companion system (POCOS), with and without an AI-based chatbot, was discussed in patient focus groups.

**Results:**

Increasingly available digital apps for fibromyalgia currently focus primarily on psychoeducational content, whereas exercise programs and multimodal concepts are less frequently integrated, despite their evidence base and patient demand. Clinical trials have demonstrated positive effects for certain online programs. Focus groups emphasized the need for personalized, low threshold and user-friendly app designs. The use of AI-powered chatbots in this context is both feasible and desired but requires well-defined content and regulatory frameworks. On a diagnostic level, machine learning can be used to identify distinct fibromyalgia types, which can support personalized treatment.

**Discussion:**

The digital treatment of fibromyalgia is both feasible and effective but requires consistent evidence-based implementation, active participation of people with fibromyalgia, technical interoperability and a sensitive design approach tailored to this heterogeneous patient population.

Fibromyalgie ist ein komplexes chronisches Schmerzsyndrom, das Betroffene körperlich, psychisch und sozial stark belastet. Klassische medikamentöse Therapien zeigen oft nur begrenzte Wirkung, weshalb Selbstmanagementstrategien wie Bewegung, kognitive Verhaltenstherapie und Achtsamkeit im Fokus stehen. Digitale Anwendungen und KI(künstliche Intelligenz)-gestützte Chatbots eröffnen neue Möglichkeiten, diese evidenzbasierten Ansätze flexibel, personalisiert und ortsunabhängig in den Alltag der Betroffenen zu integrieren.

Die Fibromyalgie ist ein chronisches Schmerzsyndrom, das durch generalisierte muskuloskeletale Schmerzen, ausgeprägte Fatigue, nichterholsamen Schlaf sowie kognitive und vegetative Begleitsymptome gekennzeichnet ist [1]. Typisch ist das dauerhafte Vorliegen funktionell gestörter Körpersysteme, insbesondere des autonomen Nervensystems [2]. Dabei bleiben diese meist ohne pathologisches Korrelat in der Blutanalyse, den radiologischen Untersuchungen oder anderen diagnostischen Abklärungen.

Die Diagnosestellung selbst erfolgt häufig mit erheblicher Verzögerung: Im Durchschnitt vergehen über 2 Jahre, und es werden mehr als 3 (fach)ärztliche Konsultationen in Anspruch genommen, bevor die Diagnose gestellt wird [3]. Psychische Faktoren spielen bei der Fibromyalgie eine wichtige Rolle, insbesondere im Rahmen von Angststörungen, Depressionen, Katastrophisierung, Alexithymie (Gefühlsblindheit) oder einer posttraumatischen Belastungsstörungen [4]. Auffallend ist bei der Fibromyalgie das lange Bestehen einer Schmerzsymptomatik – bei vielen Patient:innen seit der Kindheit oder Adoleszenz, z. B. in Form von Wachstumsschmerzen, Migräne oder Bauchschmerzen [5]. In den meisten Fällen liegt ein noziplastischer Schmerztyp mit einer Hypersensibilität des zentralen Nervensystems vor, die in der funktionellen Magnetresonanztomographie (MRT) oder auch Positronenemissionstomographie (PET) von Gehirn und Rückenmark nachweisbar ist [6, 7]. Experimentell zeigen sich zudem erhöhte Konzentrationen erregender Neurotransmitter wie Glutamat im Kortex und in der Schmerzmatrix [8].

## Behandlung – pharmakologisch versus nichtpharmakologisch

Die Wirksamkeit medikamentöser Therapien bei der Fibromyalgie ist begrenzt. Metaanalysen zeigen nur geringe Effektstärken, etwa für Amitriptylin, Duloxetin oder Pregabalin, insbesondere im Hinblick auf Schmerzreduktion, Schlaf oder Fatigue. Zudem treten unerwünschte Nebenwirkungen bei Fibromyalgiepatient:innen erfahrungsgemäß häufiger auf [9]. Entsprechend empfehlen die überarbeiteten Leitlinien der European League Against Rheumatism (EULAR) ein stufenweises Vorgehen, bei dem zunächst nichtmedikamentöse Maßnahmen im Vordergrund stehen [10]. Im Zentrum stehen dabei patientenzentrierte Aufklärung, angeleitetes körperliches Training sowie psychologische Verfahren wie die kognitive Verhaltenstherapie. Bei schwerer Beeinträchtigung wird ein multimodales Rehabilitationsprogramm empfohlen [11].

## Herausforderungen

Die praktische Umsetzung dieser Empfehlungen zur multimodalen Therapie der Fibromyalgie ist mit Herausforderungen verbunden. Der Zugang zu spezialisierten Programmen ist regional ungleich verteilt, die Gesundheitsversorgung steht unter erheblichem Ressourcendruck, und viele Betroffene fühlen sich trotz Diagnose nicht ausreichend versorgt und schon gar nicht verstanden. Zwar erhält ein Teil der Patient:innen mit Fibromyalgie eine psychologische oder psychiatrische Begleitung (mit oder ohne psychotrope medikamentöse Behandlung), jedoch findet oft kein Austausch zwischen psychischen und somatischen Disziplinen statt. Hinzu kommen Belastungssituationen im familiären Umfeld und insbesondere am Arbeitsplatz – nicht selten mit langen Arbeitsausfällen, Burn-out oder dauerhafter Arbeitsunfähigkeit. Parallel dazu fühlen sich Patient:innen im Stich gelassen und flüchten in Passivität und Resignation. Der inadäquate Einsatz von (starken) Opiaten, der explizit nicht empfohlen wird, führt zur Abhängigkeit oder sogar zu einer paradoxen Schmerzverstärkung. Auch andere iatrogene Faktoren wie unnötige Operationen oder Rückeninfiltrationen können letztendlich schmerzverstärkend wirken.

Auch bei entzündlich rheumatischen Erkrankungen kann es zur Entwicklung einer Fibromyalgie kommen, insbesondere bei der (HLA-B27-negativen) Spondyloarthritis oder beim Sjögren-Syndrom leiden bis zu 40 % der Patient:innen zusätzlich an einer Fibromyalgie [12]. Dies führt zu gehäuften Therapiewechseln oder -abbrüchen aufgrund unzureichender Wirksamkeit oder Unverträglichkeit. Dabei ist zu beachten, dass Krankheitsaktivitäts-Scores wie der DAS (Disease Activity Score) stark von subjektiven Symptomen – insbesondere Schmerz – beeinflusst werden und bei gleichzeitiger Fibromyalgie nachweislich höher ausfallen [13].

## Fibromyalgietypen und digitale „Personas“

Digitale Applikationen kommen nicht erst als Apps für Patienten, sondern idealerweise bereits viel früher zum Einsatz, z. B. bei der Einteilung von Fibromyalgietypen. Zwar ist die klinische Präsentation der Fibromyalgie meist ähnlich: ein erhöhter Symptom Severity Score und/oder Widespread Pain Index sowie erfüllte ACR(American College of Rheumatology)-2010- oder FiRST-Kriterien [14]. Letztere sind insbesondere bei einer assoziierten Fibromyalgie im Kontext entzündlich rheumatischer Erkrankungen sinnvoll, da sie weniger auf „widespread pain“ (das auch z. B. infolge von Enthesitiden auftreten kann) beruhen als die ACR-Kriterien [15]. Nicht alle Patient:innen zeigen aber eine ausgeprägte Widespread-pain-Komponente; bei vielen liegt eher ein regionales Schmerzsyndrom vor, z. B. als chronischer Rückenschmerz. Andere wiederum berichten primär über ausgeprägte Müdigkeit und Erschöpfung. Hier besteht eine deutliche Überlappung mit dem Chronic-Fatigue-Syndrom. Auch zum Long-COVID-Syndrom bzw. anderen postviralen Erkrankungen bestehen zahlreiche Gemeinsamkeiten. Viele der Long-COVID-Patient:innen erfüll(t)en die Fibromyalgiekriterien und haben nicht selten bereits vor der Infektion eine Fibromyalgiediagnose erhalten [16].

Die Bestimmung von Fibromyalgiesubtypen oder Cluster wurde in einer kürzlichen Metaanalyse durchgeführt. Mithilfe von maschinellem Lernen wurden hier verschiedene Fibromyalgieprofile deutlich, insbesondere hinsichtlich Schwere der Symptomatik, Schmerzen vs. Fatigue, adaptiv vs. maladaptivem Coping sowie dem unterschiedlichen Ansprechen auf Therapien inklusive multimodaler Behandlung [17]. Allerdings waren die klinischen Variablen eingeschränkt, es wurden in erster Linie der Fibromyalgia Impact Questionnaire (FIQ) sowie Schmerz- und Müdigkeits-Scores analysiert. In einer eigenen Studie mittels „unsupervised machine learning“ konnten wir 5 unterschiedliche Schmerztypen im Rahmen eines multimodalen Schmerzprogramms mit deutlich mehr klinischen Variablen identifizieren, darunter auch Schlaf, Beruf, Herkunft etc. In einem zweiten Schritt wurden diese „Personas“ mithilfe generativer KI (OpenAI) visualisiert [18]. Dies hatte einen Einfluss auf die Entwicklung eines eigenen Online-Therapieprogramms, was im Abschnitt „User experience“ noch erläutert wird. Als häufigster Phänotyp bzw. „Persona“ zeigten sich übergewichtige, kinesiophobe Frauen in der Peri- oder Postmenopause, charakterisiert durch Schlafstörungen, Depressionen und Brain Fog, häufig begleitet von unspezifischen Entzündungen wie aktivierten Arthrosen, Bursitiden oder Sehnenverkalkungen. Die zweithäufigste Persona ist durch Betroffene im Alter von 20 bis 35 Jahren charakterisiert mit Hypermobilität, seit Kindheit oder Adoleszenz bestehender Migräne sowie einem psychischen oder physischen Trauma in dieser Lebensphase. Auf die weiteren Personas wird hier nicht eingegangen. Es wird aber deutlich, dass z. B. für Persona 1 metabolische hormonelle Faktoren im Vordergrund stehen und im Rahmen des Selbstmanagements modifiziert werden (körperliche Übungen, Gewichtsverlust bzw. antientzündliche Ernährung, kognitive Verhaltenstherapie [KVT]). Andererseits liegen bei Persona 2 mitunter tiefer gehende psychische Konflikte zugrunde und können z. B. als Traumatherapie online behandelt werden; außerdem kann die Hypermobilität durch spezifische Übungen adressiert werden.

## Selbstmanagement bei Fibromyalgie

Selbstmanagement ist ein zentraler Bestandteil der evidenzbasierten Behandlung der Fibromyalgie [19]. Es umfasst Maßnahmen, die Patient:innen eigenverantwortlich durchführen, um ihre Symptome zu bewältigen, ihre Funktionsfähigkeit zu verbessern und ihre Lebensqualität zu erhöhen. Zu den wichtigsten Strategien gehören regelmäßige körperliche Aktivität, KVT, Achtsamkeit, Entspannungstechniken und die strukturierte Vermittlung von Wissen über die Erkrankung (Psychoedukation). Die EULAR-Leitlinie von 2017 empfiehlt insbesondere körperliches Training; hier werden Ausdauer- und Krafttraining hervorgehoben, die Schmerzen, Fatigue, Schlaf, Depression und funktionelle Einschränkungen bei der Fibromyalgie verbessern können [19]. Aerobes Training zeigt besonders Effekte auf Schmerz und Lebensqualität, während Krafttraining zusätzlich Fatigue und Schlaf positiv beeinflusst. Dehn- und Beweglichkeitsübungen hingegen werden nur schwach oder gar nicht empfohlen, da die Evidenzlage unzureichend ist. Insgesamt gilt Bewegung als einer der wirksamsten Bestandteile des Fibromyalgieselbstmanagements.

Zu den wichtigsten Strategien gehören KVT und Bewegungstherapie

KVT und Gesundheitsschulungen werden in allen Leitlinien zur Behandlung der Fibromyalgie empfohlen [10]. Sie stärken die Selbstwirksamkeit und helfen, negative Gedanken und Verhaltensmuster zu reduzieren. KVT kombiniert edukative Inhalte zur Schmerzphysiologie mit Strategien wie kognitiver Umstrukturierung, Aktivitätsdosierung, Problemlösung, Schlafhygiene und Hausaufgaben zur Anwendung im Alltag. Studien zeigen, dass die KVT Schmerzen, Stimmung und Funktion sowohl kurzfristig als auch längerfristig (bis zu 12 Monate) verbessern kann, auch wenn die Effektstärken meist klein sind [20]. Erweiterungen von KVT umfassen u. a. die Akzeptanz- und Commitment-Therapie (ACT), die in den unten aufgeführten Apps bzw. klinischen Studien besonders genutzt wird. ACT zielt auf Achtsamkeit, Akzeptanz und wertegeleitetes Handeln ab und zeigt laut Metaanalysen moderate bis gute Effekte auf Lebensqualität, Funktionsfähigkeit und psychisches Wohlbefinden. Zwischen Mindfulness und ACT gibt es keinen Unterschied [21]. Edukative Ansätze wie die „Pain Neuroscience Education“ (TPNE) verbessern Wissen, Angst vor Bewegung, Katastrophisieren, Schmerzen und Funktionsniveau teils bis zu einem Jahr nach der Intervention [22]. Weitere edukative Inhalte betreffen Alltag, Ernährung, Sexualität und soziale Strategien.

Insgesamt sollten edukative und psychologische Ansätze integraler Bestandteil eines multimodalen (auch digitalen) Behandlungsplans sein, nicht als Einzelmaßnahme, sondern im Zusammenspiel mit Bewegung und anderen aktiven Therapien. Multimodale Programme, die Bewegung, Psychotherapie und Edukation kombinieren, zeigen in Studien die besten Ergebnisse hinsichtlich Schmerzreduktion, Fatigue und funktioneller Verbesserung. Selbstmanagementinterventionen führen auch langfristig zu einer besseren Krankheitsbewältigung und verringerten Inanspruchnahme medizinischer Leistungen und fördern das Gefühl von Kontrolle, Autonomie und Selbstwirksamkeit als zentrale Faktoren für den Umgang mit einer chronischen Schmerzerkrankung wie der Fibromyalgie. Die Wirksamkeit hängt maßgeblich von Motivation, Verständlichkeit, Anwendbarkeit und v. a. Adhärenz im Alltag ab, was besonders bei der digitalen Form der Behandlung zum Tragen kommt [23].

## Digitale Behandlungsstrategien

Digitale Therapie bezeichnet den Einsatz digitaler Gesundheitsanwendungen, insbesondere Apps, Wearables oder webbasierten Programmen, zur Unterstützung der Diagnose, Behandlung oder Krankheitsbewältigung. Die bislang erhältlichen Apps für Fibromyalgie dienen fast ausschließlich Letzterem, d. h. dem Selbstmanagement. Diese Apps sind für chronische Schmerzsyndrome wie die Fibromyalgie als „Stand-alone“-Online-Programme erhältlich oder Coach-basiert mit regelmäßigem Austausch mit einem menschlichen Therapeuten.

Digital-Health-Apps werden in Deutschland als Software nicht über die Hilfsmittelliste erstattet, sondern separat über das DiGA(digitale Gesundheitsanwendung)-Verfahren des BfArM (Bundesinstitut für Arzneimittel und Medizinprodukte) erstattungsfähig gemacht. In Deutschland gewinnen DiGAs, wenngleich langsamer als angenommen, an Bedeutung [24, 25]. Für die Zulassung müssen DiGAs nachweisen, dass sie eine positive Versorgungsauswirkung haben, etwa durch Symptomlinderung, Verbesserung von Funktionsparametern oder patientenrelevante Struktur- und Verfahrensverbesserungen. In Bezug auf Fibromyalgie steht momentan nur HelloBetter Chronischer Schmerz als DIGA spezifisch für die Fibromyalgie zur Verfügung, Selfapy ist nicht mehr zugelassen. Jedoch sind einige Anwendungen für chronische Schmerzen allgemein (z. B. Vivira, Zanadio bei Adipositas mit Schmerzkomponente) verfügbar (https://diga.bfarm.de). Diese Anwendungen bieten oft eine Kombination von verhaltenstherapeutischen Modulen, Achtsamkeitstrainings, Bewegungstraining und Tagebuchfunktionen.

Für die Zulassung müssen DiGAs nachweisen, dass sie eine positive Versorgungsauswirkung haben

In einer Real-World-Studie wurden in Deutschland verschriebene DiGAs untersucht [25]. Nur 15 % der User:innen haben das DIGA-Programm vollständig zu Ende geführt. Der deutlichste Effekt von DiGAs war in Bezug auf Erschöpfung und Gewichtsmanagement zu beobachten. DiGA-spezifisch profitierten insbesondere Patient:innen mit Rückenschmerzen und Schlaflosigkeit. Hingegen konnte kein signifikanter Effekt auf Patientenaktivierung, Gesundheitskompetenz, Schmerz, allgemeinen Gesundheitszustand oder Krankheitsaktivität nachgewiesen werden, was methodische Fragen oder Fragen der Patientenselektion aufwirft.

Die Tab. 1 zeigt eine Übersicht über aktuell verschiedene verfügbare Fibromyalgie-Apps gemäß Suche im DiGA-Verzeichnis, in App-Stores und Google. Inhaltlich liegt der Schwerpunkt überwiegend auf psychoedukativen Inhalten, insbesondere auf der KVT, ACT und der „pain neuroscience education“. Inhaltlich entsprechen die digitalen Ansätze also dem empfohlenen nichtdigitalen Selbstmanagement. Von hier 11 ausgesuchten Apps enthielten 8 therapeutische Module, meist in Form von strukturierten Lektionen, interaktiven Übungen oder virtuellem Coaching. Körperliche Aktivität, etwa durch Anleitung zu Ausdauer- oder Krafttraining, wurde in 6 Apps integriert, häufig über Videos, Bewegungstagebücher oder auch Anbindung an Wearables. Hier ist die Anwendung zu nennen, welche über eine App mit einem tragbaren transkutanen elektrischen Neurostimulator (TENS) am Bein regelt und hierdurch sensible Hautnerven innerviert (Quell, NeuroMetrix, USA). Zwar war die Studie im primären Endpunkt negativ („patient global impression of change“ [PGIC]), aber bei Patient:innen mit höherer Schmerzempfindlichkeit wurde ein signifikanter Unterschied nachgewiesen, was auch zur De-novo-Zulassung der FDA (Food and Drug Administration) führte [26]. Mind-Body-Techniken wie Meditation, Atemübungen und Achtsamkeit fanden sich in etwas weniger als der Hälfte der Anwendungen. Rund die Hälfte der untersuchten Apps kombiniert mindestens 2 der 3 evidenzbasierten Selbstmanagement-Interventionsbereiche (Psychoedukation, Bewegung, Mind-Body) und ist somit als multimodal einzustufen. Zu diesen zählen die zertifizierten DiGAs HelloBetter Chronischer Schmerz (HelloBetter, Hamburg) und Selfapy Chronischer Schmerz (Selfapy, Berlin) sowie die US-amerikanische App Stanza (Swing Therapeutics, USA), die als verschreibungspflichtige digitale Therapie von der FDA zugelassen wurde. Die PROSPER-FM-Studie, publiziert in *Lancet* im Jahr 2024 [27], nimmt einen besonderen Stellenwert ein. Hier wurde die Stanza-App, ein selbstgesteuertes Smartphone-Programm auf Basis von ACT, bei 275 Fibromyalgiepatient:innen in einer multizentrischen Studie in den USA untersucht. Verglichen wurde die App-basierte ACT-Therapie vs. eine aktive Kontroll-App, die Symptom-Tracking und edukative Materialien bot. Wesentliche Ergebnisse nach 12 Wochen waren:

- 70,6 % der Nutzer:innen der Stanza-Gruppe berichteten über eine Verbesserung des Allgemeinzustands (PGIC) verglichen mit 22,2 % in der Kontrollgruppe (*p* < 0,001),
- signifikante Verbesserungen in allen Kernbereichen: Revised Fibromyalgia Impact Questionnaire(FIQ‑R)-Gesamtwert, Schmerzintensität und -interferenzen, Fatigue, Schlafqualität, depressive Symptome und körperliche Funktionsfähigkeit (Effektstärke ~0,65; *p* < 0,001),
- es wurden keine behandlungsbedingten unerwünschten Ereignisse beobachtet.

**Tab. 1:** Bestehende Online-Selbstmanagementprogramme für Fibromyalgie

App-Name	Therapeutische Mechanismen	Zulassungsstatus	Hinweise
*Stanza* (Swing Therapeutics, USA)	ACT, KVT, Achtsamkeit, Tagebuch, Erinnerungen	FDA-zugelassen (USA)	12-wöchiges strukturiertes Behandlungsprogramm
*HelloBetter Chronischer Schmerz* (HelloBetter, Hamburg)	KVT, ACT, Achtsamkeit	DiGA-zertifiziert (Deutschland)	12-wöchiges strukturiertes Behandlungsprogramm
*Selfapy Chronischer Schmerz* (Selfapy, Berlin)	KVT, Achtsamkeit, Edukation	Nicht (mehr) DiGA-zertifiziert	12-wöchiges strukturiertes Behandlungsprogramm
*Manage My Pain* (ManagingLife, Kanada)	Schmerzprotokollierung, Tagebuch, Selbstbeobachtung	Verfügbar in App-Stores	Fokus auf der Erfassung von Schmerzverläufen. Kommunikation mit Behandler
*Quell Fibromyalgie* (NeuroMetrix, USA)	Neuromodulation durch Wearable (TENS), Symptomverfolgung	FDA-zugelassenes Medizinprodukt	TENS-Applikation über Manschette am Bein, steuerbar über App
*FibroMapp* (Bodymap Apps, UK)	KVT, Symptomverfolgung, Medikamentenmanagement	Verfügbar in App-Stores	Kombiniert Verlaufsdokumentation und KVT-Werkzeuge
*MoreGoodDays* (More Good Days Pty Ltd, Australien)	KVT, Achtsamkeit, Edukation	Verfügbar in App-Stores	Strukturierte KVT-Module, Lebensstilerziehung und Achtsamkeitstechniken
*PainScale* (Boston Scientific, USA)	Symptomverfolgung, KVT, Unterstützung durch die Community	Verfügbar in App-Stores	Community-orientierte Plattform mit integrierter Wissensvermittlung und Protokollierung
*FibroTrack* (FeliXGear, Ungarn)	Symptomverfolgung, Lifestyle-Management	Verfügbar in App-Stores	In Verbindung mit Garmin Uhren
*Curable* (Curable Inc, USA)	PNE, KVT, Meditation, Schreibübungen	Verfügbar in App-Stores	Umfassende App, die auf Schmerzverarbeitung und Bewältigungsstrategien abzielt
*FibroWALK* (Spanien)	KVT, Achtsamkeit, therapeutische Bewegung und PNE	Keine zertifizierte App; Bereitstellung über YouTube und E‑Mail	Multikomponentenprogramm mit wöchentlichen Videoeinheiten

*KVT* kognitive Verhaltenstherapie, *ACT* Akzeptanz- und Commitment-Therapie, *FDA* US-amerikanische Arzneimittelbehörde (Food and Drug Administration), *DiGA* digitale Gesundheitsanwendung, *mHealth* mobile Gesundheitstechnologien, *PNE* Pain Neuroscience Education, Schmerz-neurowissenschaftliche Aufklärung. *TENS* transkutane elektrische Neurostimulation

Ergänzende, aber noch nicht publizierte Beobachtungen deuten darauf hin, dass der Nutzen eines solchen Programms auch über 12 Monate erhalten bleibt: In einer 12-Monate-Follow-up-Studie blieben 79,6 % der Patient:innen verbessert und Verbesserungen hinsichtlich Schmerzen, Müdigkeit, Schlaf und FIQ-Wert stabil [28]. Viele digitale Gesundheitsstudien, darunter auch jene zu Quell und Stanza, wählen den PGIC als primären Endpunkt, da er einfach, patientenzentriert und regulatorisch anerkannt ist. Diese Wahl wirft jedoch methodische Bedenken auf: PGIC ist stark subjektiv und anfällig für Verzerrungen durch Erwartungen, Placeboeffekte oder allgemeine Zufriedenheit mit der Behandlung. Im Gegensatz zu krankheitsspezifischen Instrumenten wie dem FIQ‑R bildet PGIC keine spezifischen Symptomdimensionen wie Schmerz, Fatigue oder Funktion ab. Obwohl statistisch leichter signifikante Effekte erzielt werden können, besteht die Gefahr, den tatsächlichen Behandlungseffekt zu überschätzen. In der HelloBetter-App wurde der Multidimensional Pain Inventory – Interference Scale (MPI-Interferenzskala) als primärer Endpunkt für die Messung der Schmerzen genutzt, der spezifischer ist als der PGIC. Allerdings wurden hier nicht nur Fibromyalgiepatient:innen untersucht, und die Studien für Schmerz sind nicht endgültig veröffentlicht.

Generell ist anzumerken, dass es für DiGA-Verschreiber:innen bislang wenig übersichtlich ist, welche Art von App am besten zu den einzelnen Patient:innen passt. Im Vergleich zu pharmazeutischen Medikamenten gab es deutlich weniger – oder gar keine – Informationsveranstaltungen. Das DiGA-Register könnte hier möglicherweise noch strukturierter und edukativer gestaltet werden, um die Entscheidungsfindung und die Verschreibung selbst zu erleichtern.

## „User experience“

**Abb. 1:**
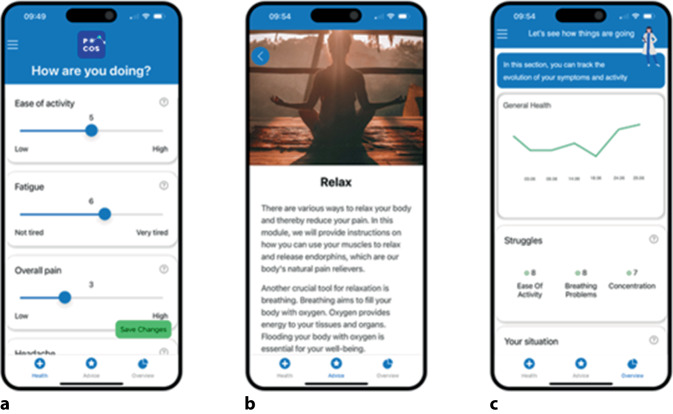
Exemplarische Fibromyalgie-App POCOS (Pain Organiser and Companion System) für Patient:innen mit Fibromyalgie, wie sie in der User-Experience-Studie eingesetzt wurde. Navigation über Leiste unten zur Symptomeingabe (**a**), Online-Therapieprogramm (**b**) und Verlaufsdarstellung (**c**)

Adhärenz und eine gute Nutzerführung sind essenziell für den Erfolg einer digitalen Therapie [23, 29, 30]. In einer eigenen Studie wurde die Nutzung einer selbst entwickelten App bei Patient:innen im Anschluss an ein 2‑wöchiges stationäres multimodales Schmerzprogramm getestet. Initial wurde es für Long-COVID-Patient:innen mit Fibromyalgiesymptomen entwickelt und im Anschluss auf chronische Schmerzsyndrome erweitert [31, 32]. Die App POCOS (Pain Organiser and Companion System) enthielt ein Online-Therapieprogramm, Symptomverfolgung und Tagebuch (Abb. 1). Von Patientenseite wurde insbesondere hervorgehoben, dass man sich nach einem Schmerzprogramm weniger allein gelassen fühlt. Wichtig war eine niedrigschwellige Zugänglichkeit, insbesondere für digital weniger versierte Patientengruppen oder Betroffene mit Depression oder Erschöpfung. Zusammenfassend lässt sich sagen: Fibromyalgiepatient:innen legten besonderen Wert auf die Einfachheit der zu benutzenden App mit klaren und verständlichen Inhalten sowie Aspekte, die ihre eigene Autonomie, Vertrauen und ihre Motivation stärken. Für sie war es entscheidend, dass die App zuverlässig und wissenschaftlich fundiert ist. Sie betonten eine freundliche, nicht bevormundende Ansprache. Kurze und leicht verständliche Module werden generell bevorzugt, diese sollten Wissen vermitteln und gleichzeitig zur aktiven Selbstfürsorge motivieren. Ebenso schätzen die Nutzer:innen hochwertige Videos, speziell physiotherapeutische Anleitungen und Module, die über ihre Klarheit und Einfachheit motivieren. Dabei war es ihnen wichtig, dass Übungen flexibel ausgewählt und an ihre Tagesform angepasst werden können.

Adhärenz und eine gute Nutzerführung sind essenziell für den Erfolg einer digitalen Therapie

Ein zentrales Anliegen war die Personalisierung der App: Die Patient:innen wünschten sich mehr Interaktivität und z. B. die Möglichkeit, Favoritenlisten anzulegen, und einen Dialog mit einem virtuellen Coach oder Chatbot, der individuell auf ihren Zustand eingeht und passende Übungen vorschlägt. Zudem hoben die User hervor, dass die App ihre Fortschritte sichtbar machen sollte, etwa durch verwertbare Verlaufsdaten, um Zusammenhänge zwischen Aktivitäten, Symptomen und Behandlung zu erkennen. Schließlich betonten sie die Notwendigkeit von Empathie, Motivation und Positivität in Design und Kommunikation, um den emotionalen Umgang mit der Erkrankung zu unterstützen. Allerdings ist die Personalisierbarkeit bei den aktuell erhältlichen Fibromyalgie-Apps sehr eingeschränkt, was u. a. mit der Regulierung bzw. Vergütung anhand der klinischen Studien zusammenhängt. Patienten vermissten teilweise Intuitivität, eine einfache Navigation und bemängelten eine fehlende Nutzerführung. Dies sind Faktoren, die gerade bei einer kognitiv und energetisch eingeschränkten Zielgruppe wie Fibromyalgiebetroffenen besonders relevant sind. Diese Rückmeldungen unterstreichen die Notwendigkeit, digitale Lösungen nicht nur technisch funktional, sondern auch barrierearm, übersichtlich und empathisch zu gestalten.

## Künstliche Intelligenz-Modelle und Chatbots in Online-Therapieprogrammen

**Abb. 2:**
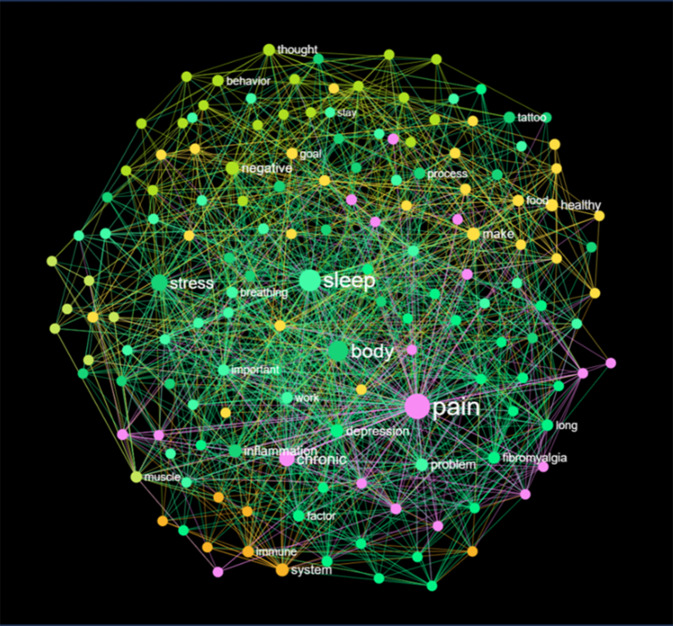
Ein Knowledge-Graph illustriert den Inhalt des Online-Therapieprogramms aus der POCOS-App (Nodus Labs)

Die Inhalte der in Tab. 1 aufgeführten Fibromyalgieprogramme sind meist nicht oder nur sehr eingeschränkt veröffentlicht. In der Regel werden lediglich die Hauptmethode, wie bei Stanza die Akzeptanz- und Commitment-Therapie, sowie die Programmdauer angegeben. Dies erschwert den Vergleich einzelner Programme und deren Anpassung an spezifische Patientengruppen. Dies könnte z. B. mithilfe von Knowledge-Graphen wie hier für POCOS (Nodus Labs) dargestellt werden (Abb. 2). Durch den Einsatz solcher Large-Language-Model(LLM)-unterstützten Knowledge-Graphen lassen sich Inhalte von Schmerzprogrammen nicht nur visualisieren und transparenter gestalten, sondern auch gezielte Gap-Analysen durchführen und inhaltliche Verbesserungen ableiten.

In einem aktuellen Projekt wurden Teile von POCOS zudem in einen LLM-basierten Chatbot (Buddy, ZTM, Deutschland) integriert und erneut von Patient:innen getestet. Dabei zeigte sich, wie wichtig eine personalisierte Nutzung von LLM-Chatbots ist. Dies bedeutet, dass der Chatbot auf Inhalte vorheriger Konversationen bzw. Prompts eingehen können muss und sich auf den Symptomtracker sowie bereits absolvierte Programminhalte beziehen sollte. Gleichzeitig wurde das Potenzial des Chatbots klar, da er viel personalisierter und tiefer gehend als bisher durch ein Online-Behandlungsprogramm führt und damit die Adhärenz sehr wahrscheinlich verbessert. Außerdem spricht für ihren Einsatz die Möglichkeit einer sprachlich empathischen oder gezielt gewählten Ansprache. Aktuell sind Chatbots in Therapieprogrammen jedoch überwiegend textbasiert, und oft ist nicht eine natürliche Sprachinteraktion optimiert. Limitationen von Chatbots sind die fehlende Systematik und Gefahr, sich in Details zu verlieren, sowie die noch unzureichende Interoperabilität mit anderen Datenquellen, z. B. Symptomverläufe oder Wearables (Mobilität, Schlaf). Hier können spezifische LLM-Modelle wie das kürzlich publizierte SensorLM, ansetzen, das Sensordaten in Text umwandelt und für Nutzer:innen interaktiv zugänglich macht [33].

KI-Chatbots können emotional unterstützen, Wissen vermitteln und zur Patientenaktivierung beitragen

Derzeit existiert keine Evidenz für die alleinige Nutzung von Chatbots in der Fibromyalgie- oder chronischen Schmerztherapie; entsprechende Studien laufen. Eine Proof-of-Concept-Studie nutzte jedoch eine LLM-gestützte Sentiment-Analyse (Mistral 7B), um subtile sprachliche Schmerzexpressionsmuster von Patient:innen zu identifizieren [34]. Fibromyalgie konnte dabei mit einer Genauigkeit von 87 % von anderen chronischen Schmerzzuständen unterschieden werden. Für chronische Rückenschmerzen zeigte eine Analyse von ChatGPT, Bard und ChatGPT 4.0, dass rund 56 % der Antworten zu Selbstmanagement- und Behandlungsfragen korrekt waren, wenngleich die Zuverlässigkeit variierte [35]. Chatbots wurden zudem zur Schmerzanamnese und -aufklärung in verschiedenen Altersgruppen als gut akzeptabel bewertet, auch wenn Schwächen bei der stimmlichen Ausdrucksweise genannt wurden [36]. Insgesamt deuten die bisherigen Ergebnisse darauf hin, dass KI-Chatbots potenziell emotional unterstützen, Wissen vermitteln und zur Patientenaktivierung beitragen können, insbesondere dann, wenn sie fachlich geprüft, gut strukturiert und in einen klaren Selbstmanagementrahmen eingebettet sind (Tab. 2).

**Tab. 2:** Arten von Digital-Health-Programmen

App-Typ	Dauer/Struktur	Intervention	Dashboard	Coach/Bot	Verlaufskontrolle	Evidenz/Vergütung	Beispiel-Apps
*Stand-alone-App*	Strukturiertes Programm (z. B. 12 Wochen)	Text, Videos, Instruktionen, KVT, Übungen	Optional	Keiner	Symptomtracking, Tagebuch	Evidenz vorhanden, z. T. DiGA	HelloBetter, Selfapy Stanza
*App mit Coach*	Flexibles oder strukturiertes Programm	Wie oben + Gespräche, z. B. transkribierte Sprachnachrichten	Ja	Coach	Tracking + Feedback durch Coach	Gute Evidenz, vergütet über Therapeut:innen	Selfapy (mit Coaching)
*App mit Zusatzgerät*	Strukturiertes Programm mit Gerät/Wearable	Instruktionen + körperliche Übungen (z. B. Rehabilitation)	Ja	Keiner	Messung via Gerät (z. B. Bewegung)	Teilweise DiGA, Evidenz gerätespezifisch	Quell Fibromyalgia
*App mit Chatbot*	Flexibles Programm mit Prompts	Wie Stand-alone-App, aber interaktiver mit Prompts	Optional	Chatbot	Semantisch, teilweise strukturiert	Noch keine Evidenz, Vergütung unklar	Noch in Entwicklung
*App mit Coach* *+* *Chatbot*	Flexibles Programm, Eskalation zum Coach	Bot führt, Coach bei Bedarf	Ja	Coach + Chatbot	Semantisch + menschliche Rückkopplung	Noch keine Evidenz, Adhärenz ↑, hohes Potenzial	Noch in Entwicklung
*Wearable*	Unbegrenzt	Körperliche Ziele, Aktivitätstracking	Ja	Keiner	Metrisch: Aktivität, Ziele	Nicht vergütet, z. B. Apple Watch	Apple Health, Fitbit
*Wearable mit Chatbot*	Unbegrenzt	Ziele, Aktivität + semantische Bot-Interaktion	Ja	Chatbot	Metrisch + semantisch	Unklar	n. a.
*Nur Chatbot*	Unbegrenzt	Semantische Text- oder Sprachinteraktion	Nein	Chatbot	Semantisch	Unklar, bisher keine RCTs	GPT-basiert, experimentell

*KVT* kognitive Verhaltenstherapie, *DiGA* digitale Gesundheitsanwendung, *RCTs* randomisiert kontrollierte Studien, *n.a.* nicht angegeben

## Schlussfolgerung

Die digitale Behandlung der Fibromyalgie ist möglich und eröffnet neue Chancen, evidenzbasierte Selbstmanagementstrategien wie kognitive Verhaltenstherapie, Bewegung und Achtsamkeit einem breiteren Patientenkreis zugänglich zu machen. Während bestehende Apps v. a. psychoedukative Inhalte bieten, sind multimodale Konzepte und Bewegungsprogramme – auch unter Einbezug von Wearables – bislang unterrepräsentiert. Erfolgreiche digitale Therapien erfordern eine patientenzentrierte, personalisierte und barrierearme Gestaltung. KI-gestützte Chatbots können hierbei unterstützen, müssen jedoch inhaltlich fundiert, sicher und interoperabel sein. Die PROSPER-FM-Studie markiert einen wichtigen Meilenstein mit signifikanten klinischen Ergebnissen. Für eine breite Implementierung sind digitale Gesundheitskompetenz bei Patient:innen und Fachpersonal, klare regulatorische Rahmenbedingungen, Datenschutz, Interoperabilität und eine angemessene Vergütung entscheidend. Digitale Ansätze sollten stets evidenzbasiert, nutzerfreundlich und in ein multimodales Versorgungskonzept eingebettet sein.

## Fazit für die Praxis

- Digitale Anwendungen können wirksame Selbstmanagementstrategien bei Fibromyalgie vermitteln und so die Versorgungslücke zwischen Diagnose und langfristiger Betreuung schließen.
- Einige Anwendungen verfügen über einen nachgewiesenen Nutzen und werden als digitale Gesundheitsanwendung (DiGA) von Krankenkassen erstattet.
- Besonders wirksam sind multimodale Konzepte, die Bewegung, Psychoedukation und Achtsamkeit kombinieren. Partizipation der Betroffenen in Entwicklung und Testung erhöht Akzeptanz und Wirksamkeit.
- KI(künstliche Intelligenz)-gestützte Chatbots können personalisierte Unterstützung bieten, müssen aber transparent, sicher und in bestehende Versorgungspfade integriert sein.
